# A novel and quick egg yolk immunoglobulin y antibody extraction method leveraging the protein liquid-liquid phase separation principle

**DOI:** 10.1016/j.psj.2025.104804

**Published:** 2025-01-11

**Authors:** Murtala Bindawa Isah, Hu Yuzhang, Mei Dang, Xiaoying Zhang

**Affiliations:** a Chinese-German Joint Laboratory for Natural Product Research, Shaanxi International Cooperation Demonstration Base, Shaanxi University of Technology, Hanzhong 723000, Shaanxi, PR China; b Department of Biochemistry, Umaru Musa Yar'adua University Katsina, Nigeria; c Centre of Molecular and Environmental Biology (CBMA), Department of Biology, University of Minho, Campus de Gualtar, Braga 4710-057, Portugal; d Department of Biomedical Sciences, Ontario Veterinary College, University of Guelph, Guelph, ON, Canada

**Keywords:** Immunoglobulin Y (IgY), Liquid-liquid phase separation (LLPS), Polyethylene glycol, Protein extraction and purification

## Abstract

This study presents a novel and efficient method for extracting immunoglobulin Y (IgY) antibodies from egg yolk based on the principle of liquid-liquid phase separation (LLPS) induced by polyethylene glycol 8000 (PEG 8000). Initial delipidation of egg yolk samples with varying PEG 8000 concentrations demonstrated optimal delipidation efficiency and protein recovery at 2.5 % PEG 8000 concentration. Subsequent IgY extraction involved inducing LLPS by raising PEG 8000 concentration to 6.5 %, resulting in turbid solutions and the formation of globular droplet-like condensates observed under a microscope. Unlike the PEG 6000 method that induced aggregation, the method developed here using PEG 8000 does not lead to the appearance of aggregates of IgY. SDS-PAGE analysis confirmed that IgY extracted was no different from the conventional PEG 6000 method, with similar purity levels (77 % vs 79 %). Enzyme-linked immunosorbent assay and western blot analysis confirmed the antigen recognition properties of the isolated IgY. This method significantly reduces the amount of PEG used, leading to substantial cost savings compared to PEG 6000. The method can be completed within one hour. Despite a slightly lower IgY yield by the method, the time- and cost-saving advantages of this method make it a promising alternative for IgY extraction in research. This proposed IgY extraction technique utilizing protein LLPS has the potential to improve the study of the physicochemical properties of IgY and optimized production, while offering a quicker and cost-effective solution for various applications in biomedical research.

## Graphical abstract

**Figure:**
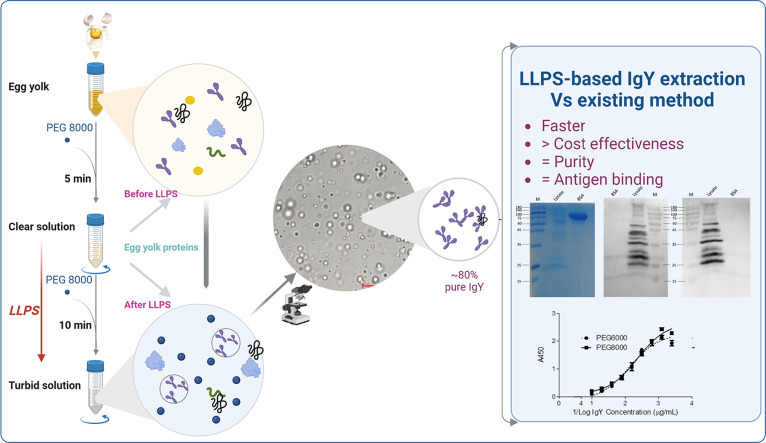

## Introduction

Immunoglobulin Y (IgY) is functionally similar to mammalian immunoglobulin G (IgG) and serve as the main serum immunoglobulin in birds, amphibians, and reptiles, transferred to egg yolk to provide passive immunity to embryos and offspring (Zhang et al., 2017). Hen egg yolk contains a substantial quantity of IgY, which can be purified at a much lower cost with high yield, fulfilling the requirements of animal welfare, better than mammalian IgG (Schade et al., 2007). Importantly, IgY exhibits several adivantages, including a stronger immune response to conserved mammalian proteins, low cross-reactivity, high stability, and no reported toxic side effects in humans and animals (León-Núñez et al., 2022). Consequently, IgY antibodies have been widely used in human and animal health as therapeutic and diagnostic tools, particularly for bacterial, viral (Grzywa et al., 2023), and parasitic (Thirumalai et al., 2019) infections. Additionally, IgY has been developed into various products such as egg yolk powder for preventing diarrhea in newborns (Rosa et al., 2015), IgY toothpaste for treating periodontitis in humans (Wang et al., 2019), and other IgY products for addressing skin disorders (Yakhkeshi et al., 2022). Therefore, IgY holds significant value for diverse aspects of biomedical research and clinical applications.

With the increasing interest in utilizing IgY for various application, there is a concurrent effort to develop more efficient and cost-effective methods for IgY isolation. Some of the earliest methods which are still in use to date, are water dilution (Akita and Nakai, 1992), polyethylene glycol 6000 (PEG 6000) precipitation (Pauly et al., 2011), ammonium sulphate (Ky and Du, 2007), caprylic acid (Deignan et al., 2000), and carrageenan method extraction methods (Tan et al., 2012). In all IgY extraction methods, the basic principle involves separating the protein component from the egg yolk lipids, followed by the selective precipitation of IgY. While organic solvents such as chloroform or phenol have been used, these methods do not yield a safe product for medical or food applications. For most industrial applications the water dilution method in combination with chromatography and filtration is more popular. For research purposes, the water dilution and PEG 6000 precipitation methods are the most commonly used, offering high yield and acceptable purity. These methods are also relatively faster and more cost-effective compared to chromatographic methods.

Recent research continues to focus on developing novel methods for IgY extraction to improve recovery, purity, scalability, speed, and cost-effectiveness. Chen et al. (2022) introduced a method for purifying IgY using common household materials and low-cost technology, designed for rapid response to viral pandemics such as COVID-19. Madera-Contreras et al. (2022) optimized the PEG 6000 extraction method developed by Pauly et al. (2011), adding an extra step to enhance IgY purity. In a different approach, Vicente et al. (2022) employed thermoresponsive aqueous micellar two-phase systems with ionic liquids, achieving a promising one-step (54 % purity) or two-step (69 % purity) IgY purification method. Similarly, Almeida et al. (2022) used aqueous biphasic systems of PEG 1000 and phosphate buffer combined with centrifugal partition chromatography, reaching up to 56 % IgY purity. More recently Su et al. (2023) reported an IgY purification method using oleic acid, while Xia et al. (2023) introduced a high-speed-shear crossflow membrane separation technique for large-scale IgY production, achieving up to 96.9 % purity. Collectively, these advancements underscore the ongoing pursuit of efficient and versatile IgY purification methods for diverse applications.

The various IgY extraction methods leverage the physicochemical and structural properties of proteins. Consequently, the development of novel IgY extraction strategies, and by extension, the extraction of other proteins, goes hand in hand with advances in understanding the behavior of proteins in solution. In the last decade, studying the liquid-liquid phase separation (LLPS) behavior of proteins have attracted increasing interest due to its involvement in various diseases (Wang et al., 2021). LLPS refers to the spontaneous separation of proteins, nucleic acids, or other macromolecules in solution into a dense phase resembling a liquid droplet. Under varying conditions, proteins separate into a protein-poor light phase and a protein-rich heavy phase, visible under a light microscope, thus can be easily traced (Alberti et al., 2019). Beyond the interest on LLPS for its role in physiology, it has also emerged as a novel way to study the biophysical properties of proteins in solution (Wang et al., 2021). Nowadays, protein aggregation can be studied under LLPS with its characteristic reversibility, separate from other forms of protein condensation, including crystallization, colloidal aggregation, and gelation, which often lead to misfolding or are not easily reversed (Wang et al., 2014).

While investigating the LLPS properties of IgY, we realized that the principle could be applied to IgY extraction and PEG 8000 could serve as an efficient polymer for such purpose. Consequently, this study aimed to develop an efficient method of extracting IgY from egg yolk by utilizing its LLPS induced by PEG 8000.

## Materials and methods

### Optimization of PEG 8000 Concentration for IgY Extraction from Egg Yolk

Yolks from six eggs were obtained, pooled together, mixed thoroughly and 10 mL portions were prepared. PEG 8000 (32 %) masterbatch was prepared by dissolving PEG 8000 in distilled water (w/v). Egg yolk solutions were mixed with different dilutions of PEG 8000 (2.25 %, 3 %, 3.75 %, and 4.5 %) in a 1:2 volume ratio at room temperature to yield final PEG 8000 concentrations of 1.5 %, 2 %, 2.5 %, and 3 %. The tubes were mixed for 10 min at room temperature on a cylinder mixer at 80 revolutions/min, followed by centrifugation at 12000 g; 4 °C for 10 min. The supernatants were collected, filtered through Whatmann No. 1 filter paper and visually compared for delipidation efficiency. Protein concentrations were estimated followed by sodium dodecyl sulphate polyacrylamide gel electrophoresis (SDS-PAGE) analysis. The filtrate with the best combination of delipidation and total protein content was processed further. Different dilutions of PEG 8000 (9 %, 11 %, 13 %, and 15 %) were added (1:1 v/v) at room temperature to give final PEG 8000 concentrations of 4.5 %, 5.5 %, 6.5 %, and 7.5 %, respectively. The tubes were mixed for 5 min on a cylinder mixer at 30 revolutions/min followed by centrifugation at 12000 g; 4 °C for 10 min. The precipitates were resuspended in 5 mL phosphate buffered saline (10 mM, pH 7.4) and the soluble IgY was stored at −20 °C. Additionally, the conventional PEG 6000 extraction method (Pauly et al., 2011) was performed in parallel for comparison.

### Microscopic observation

Double concave microscope slides and coverslips were rinsed with ddH_2_O and dried. IgY solution (20 μL) was transferred on to the slides, covered and observed using a Zeiss Axioscope 5 light microscope equipped with 40 × objective lens (NA 0.65). Zen Blue software (Carl Zeiss, Thornwood, NY, USA) was used to acquire and process images, which were exported and analyzed with Image J (Version 1.51) (Schindelin et al., 2012).

### Protein quantification

Bradford assay (Bradford, 1976) was used for the estimation of protein concentration of samples using bovine serum albumin (BSA) as the standard.

### Immunoblotting

Bacterial lysates were separated on a 12 % Laemmli SDS-PAGE gels. The proteins on the gel were transferred onto nitrocellulose membrane (Immobilon-NC, Solarbio, China) for Western blotting using a BioRad Mini Trans-blot blotter (Bio Rad, Hercules, CA, USA) set at 100 V for 2 h. After blotting, the membranes were blocked with a 5 % bovine serum albumin (BSA) in phosphate buffer saline containing 0.05 % (v/v) Tween-20 (PBST) at 4 °C for 16 h. The blocking solution was discarded and the membrane washed three times using PBST for 5 min each. The membrane was incubated with 200 μg/mL of the isolated IgY prepared in 0.5 % BSA PBST for 2 h at room temperature. After washing, the membrane was incubated in horse raddish peroxidase (HRP)-conjugated rabbit anti-chicken IgY solution (Abcam, Cambridge, MA, USA) diluted 1:6000 in 0.5 % BSA PBST and incubated at 37 °C for 1 h. The membrane was washed as before and the blots were developed by incubating the membrane in Beyotime ECL moon B chemiluminescence substrate solution following the manufacturer's instructions (Beyotime Biotechnology, Shanghai, China). Images were captured using MiniChemi® chemiluminescence imaging system (Sin Sage Technology Co., Ltd., Beijing, China).

### Enzyme-linked immunosorbent assay

To compare the affinities of the isolated IgY, ELISA was performed. Wells of polystyrene plates (Corning®, Alfred Road, Kennebunk ME 04043 USA) were coated (4°C; 16 h) with 100 µL of an antigen solution made from whole cell suspension of *Staphylococcus epidermidis* in carbonate buffer (pH 9.6). Negative control wells were coated with buffer only. The coating solution was discarded and the unoccupied sites in wells were blocked with 200 μL of blocking solution of 5 % (w/v) non-fat dry milk in phosphate buffer saline containing, 0.05 % (v/v) Tween-20 (PBST) for 2 h at 37°C. The wells were washed three times with PBST. The wells were incubated for 1 h at 37°C with 100 μL/well of a serial dilution of the isolated IgY diluted in PBST containing 2 % BSA. The wells were washed followed by incubation with 100 μL/well of 1:5000 dilution of rabbit anti-chicken IgY HRP conjugate secondary antibody (Abcam, Cambridge, MA, USA) in 2 % non-fat dry milk in PBST for 1 h at 37°C. After washing, substrate solution of 3,3′,5,5′-tetramethylbenzidine was added to the plates was added (100 μL/well) and incubated for 15 min at 37°C in the dark for color development, after which 50 μL/well 2 M H_2_SO_4_ stopping reaction was added. Absorbance values were recorded at 450 nm with an ELISA plate reader (BioTek Instruments Inc., Highland Park, VT, USA). The non-linear regression curves for antibody-antigen binding were plotted and EC50 values calculated using GraphPad Prism version 8.0.1 for Windows, GraphPad Software, Boston, Massachusetts USA, www.graphpad.com.

### Statistical analysis

Analysis of variance (ANOVA), Student's t-test, non-linear regression and Mann-Whitney U test were performed using GraphPad 8.1 for Windows, GraphPad Software, Boston, Massachusetts USA, www.graphpad.com.

## Results and discussion

### Isolation of egg yolk IgY based on LLPS principle

LLPS in proteins is caused by mechanisms including multivalent weak interactions between intrinsic disorder regions (IDR) of proteins and multivalent specific interaction networks formation by scaffold proteins (Yang et al., 2023). Under conditions for which LLPS is energetically favorable, the driving force is the exchange of intramolecular interactions within macromolecules and within water molecules for macromolecule/water interactions (Alberti et al., 2019). Thus, LLPS formation is dependent upon the strength of “native” attractive interactions between proteins. While under normal physiological conditions most proteins do not undergo LLPS, modifying salt concentration, temperature, and pH of the solution or by adding nonionic protein precipitants can promote aggregation. This phenomenon has emerged as a promising tool for protein study with potential for various applications (Wang et al., 2014). For instance, LLPS of antibodies has the potential in producing more efficient systemic delivery systems for antibody drugs, by rationally inducing LLPS to retain antibody activity (Bramham et al., 2021). Previous studies on IgG have indicated the applicability of using PEG or arginyl-glutamate to induce LLPS (Bramham et al., 2021; Wang et al., 2014). Both studies demonstrated that the binding properties of the antibodies were not affected by crowding induced by the precipitants. Thus, for IgY antibody, with its immense promise of abundance in egg yolk, these precipitants are invaluable tools for the extraction and subsequent biophysical study of IgY.

**Fig. 1:**
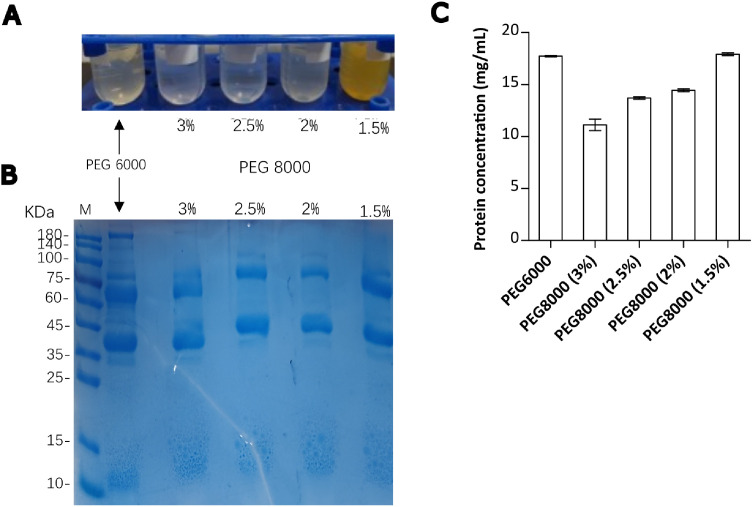
**Effectiveness of PEG 8000 in the delipidation of egg yolk. Notes: (A)** Appearance of filtrates after delipidation of egg yolks using PEG. The delipidation effect increases as the color in the tube becomes clearer. **(B)** 12 % SDS-PAGE analysis of degreased IgY yolk samples. M: Prestained protein marker (Shanghai Shengen Biotech.). **(C)** Total protein concentration in the filtrate of degreased yolk samples**.** Data are presented as the mean values from three technical repeats ± SD.

While studying the LLPS of IgY, it became evident that PEG 8000-induced IgY aggregation could be an efficient method for IgY extraction. We initially optimized the amount of PEG 8000 necessary to achieve efficient removal of egg yolk lipids and pigments and enriching the protein fraction (Fig. 1A-C). Clearer filtrates were obtained with PEG 8000 concentrations of 2 %, 2.5 %, and 3 %. These filtrates were clearer than the filtrate obtained with the use of 3.5 % PEG 6000 (Fig. 1A). Notably, a sharp decrease in protein concentration was observed at a final PEG 8000 concentration of 3 % compared to lower concentrations (Fig. 1C), indicating that this concentration is the threshold where more proteins are lost into the lipid-rich precipitate. Conversely, PEG 8000 at 2 % and 1.5 % resulted in yellowish filtrate, suggesting the presence of egg yolk pigments. This established 2.5 % PEG 8000 as an optimum concentration for the delipidation of egg yolk. Additionally, SDS-PAGE analysis revealed that, in general, PEG 8000 also removes many higher molecular weight proteins in addition to the effect (Fig. 1B).

**Fig. 2:**
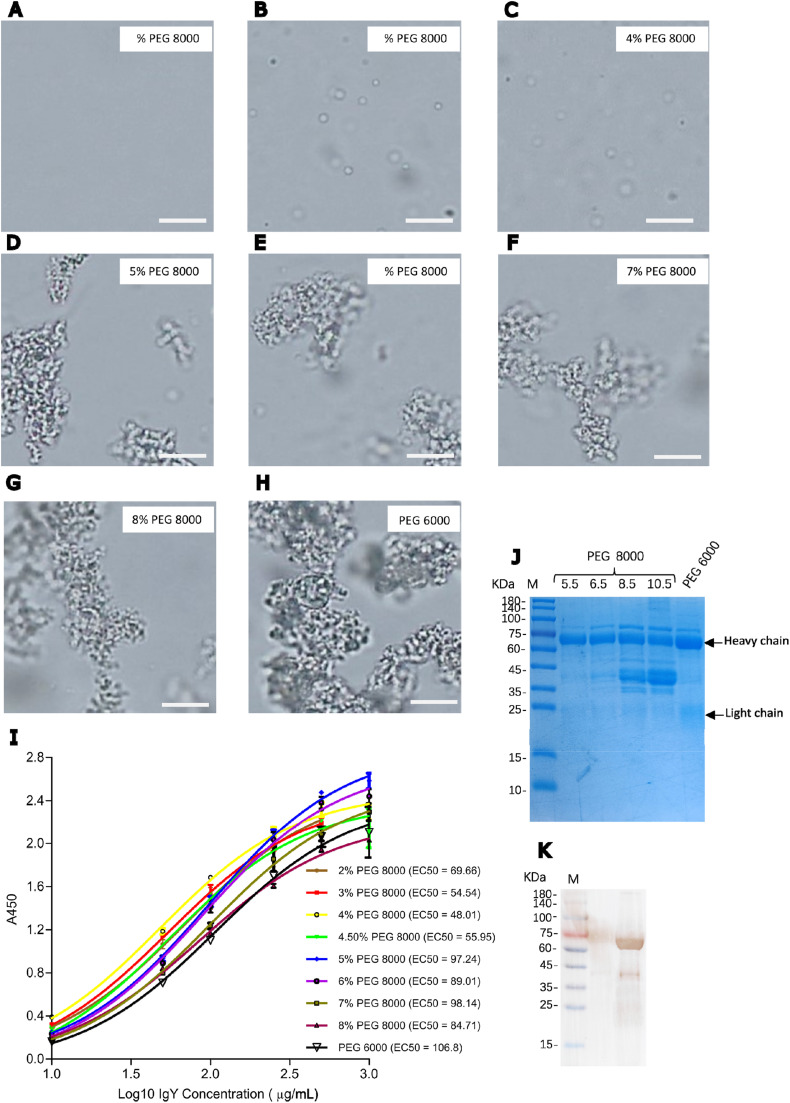
**PEG 8000 induced LLPS and purification of egg yolk IgY. Notes:** After the addition of PEG, condensates could be observed under a light microscope (**A-H**). Scale bar = 5 μm. **(I)** ELISA titration curve comparing the antigen biding properties of IgY extracted with various concentrations of PEG. The coating antigen for the ELISA was whole cells of ***Staphylococcus epidermidis***. Serial dilutions of the IgY (1000 – 10 μg/mL) were prepared and used as the primary antibodies and detected using HRP-conjugated rabbit anti-chicken IgY secondary antibody. (**J**) 12 % SDS-PAGE analysis of proteins obtained after second PEG 8000 addition after delipidation and as well as PEG 6000 precipitation. M: Prestained molecular weight marker (Shanghai Shengen Biotech.). The bands corresponding to the heavy chain (65-70 kDa) and light chain (25 kDa) of IgY are marked. (**K**) Western blot analysis of IgY purified using PEG 8000. Purified IgY separated on an SDS-PAGE gel was transferred onto nitrocellulose membrane, probeb with rabbit anti-chicken IgG HRP conjugate and visualized using 3,3′,5,5′-Tetramethylbenzidine (TMB) substrate.

The filtrate after delipidation with 2.5 % PEG 8000 yielded a balance of delipidation efficiency and protein recovery, making it suitable for subsequent IgY extraction. In the second step, LLPS was induced by adding various concentrations of PEG 8000 to raise the effective polymer concentration in the filtrate to 4.5 %, 5.5 %, 6.5 %, 7.5 %, 8.5 %, 9.5 % and 10.5 % (Fig. 2A-H). Globular droplet-like condensates of increasing size were microscopically visible, and formed aggregates at concentrations above 6.5 % (Fig. 2D-G). The PEG 6000 also induced the formation aggregates by the last PEG addition (Fig. 2H). PEG induces protein precipitation by exerting a depletion force on the protein, a property that increases with the increasing molecular weight of PEG (Annunziata et al., 2002). The depletion forces increases the intermolecular interaction among proteins in solution, irrespective of the sequence or sizes of the proteins (Poudyal et al., 2023). At low concentrations, PEG leads to LLPS while at higher concentrations the proteins tend to form aggregates, which at the extreme crowding condition and longer storage times, could result in loss of activity; which in the case of antibodies, is the loss of antigen recognition (Wang et al., 2007). To test this effect, the pellets obtained after the second centrifugation step, containing IgY, were resuspended, and the affinities of the extracted IgY to antigens was tested using a titration ELISA. The results showed that all PEG 8000 concentrations have led to IgY extraction with similar antigen binding properties, but with the lower concentrations (4.5 %−6.5 %) leading to lower EC_50_ values compared to the higher concentrations (7.5 %−10.5 %) (Fig. 2I). SDS-PAGE analysis demonstrated that the protein profile and IgY purity (estimated from the intensity of the bands corresponding to the heavy and light chains of IgY) of the products of 5.5 % and 6.5 % PEG 8000 extraction was comparable to the PEG 6000 method. Contrarily, some prominent bands that are not the size of either chain of IgY appeared in the samples resulting from extraction with higher PEG 8000 concentration (Fig. 2J). Taken together, these results seem to suggest that the lower PEG 8000 concentrations led to LLPS of IgY without causing aggregate formation, although at the expense of high protein recovery.

### The purity and affinity of the isolated IgY is comparable to an existing method

**Fig. 3:**
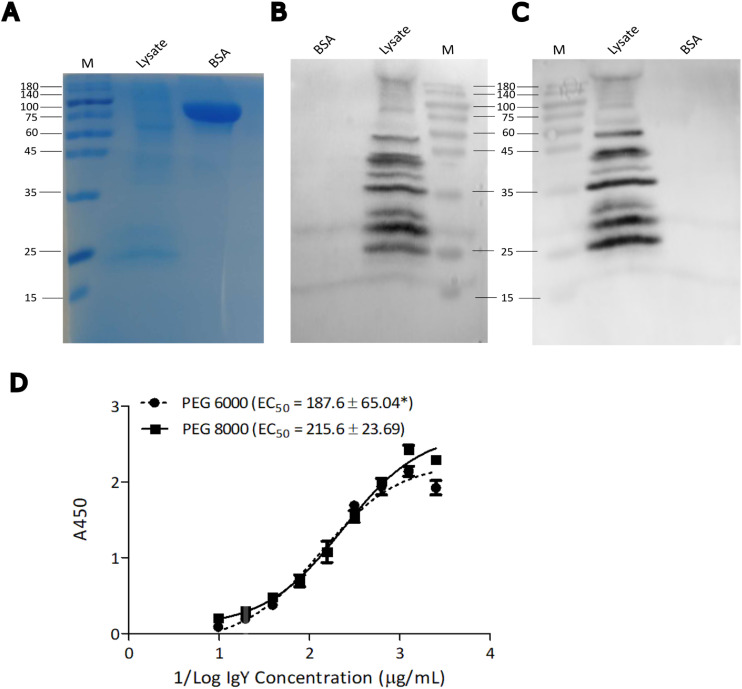
**Affinity of IgY isolated using novel PEG 8000 method. Notes:** The affinity of the IgY to antigens was assessed using Western blot analysis. Lysates of *Staphylococcus epidermidis* were separate on 12 % reducing SDS-PAGE gels and stained (**A**) or transferred onto nitrocellulose membranes. The IgY isolated using the PEG 8000 (**B**) or PEG 6000 (**C**) methods were used as primary antibodies. The blots were visualised using Chemiluminiscence subtrate (Beyotime, Inc, China). **(D)** Titration ELISA using the extracted IgY using the novel PEG 8000 method compared with the commonly used PEG 6000 purification method. The experiment was repeated three times and the EC_50_ values calculated from the non-linear regression of the curves were compared using Whitney-Mann U test. *The difference between the EC_50_ values for the two isolation methods was not Statistically significant (*p* > 0.05).

Image J analysis indicated that the purity of the IgY solution extracted at a final PEG 8000 concentration of 6.5 % was approximately 77 %, similar to the purity of IgY obtained with PEG 6000 (approximately 79 %) (Table 1). The affinities of the IgY extracted using this amount of PEG 8000 were further assessed (Fig. 3A-D). In a western blot, IgY extracted using both methods recognized similar prominent bands (Fig. 3B & C) in a lysate of the bacteria (Fig. 3A) used to immunize the hens from which the eggs were obtained. There was no statistically significant difference (*p* > 0.05) between the EC_50_ values calculated for the IgY isolated by the two methods (Fig. 3D). Thus, these results confirm that PEG 8000 is also an efficient polymer for IgY extraction and could provide an advantageous alternative to the more popular PEG 6000 extraction method.

**Table 1:** IgY yield from egg yolk by PEG 8000 extraction method compared with PEG 6000 method.

Method	Purity (%)	Total protein recovery (mg)	Total IgY recovery (mg)	IgY yield (mg/mL yolk)	Approximately PEG amount (per 15 mL yolk) (g)	PEG used (g/mg IgY yield)	Relative cost saving/15 mL yolk*	Time
PEG 8000	77 ± 2.70	49 ± 0.7	37.96	3.80	3.82	1.01	40 %	<1 h
PEG 6000	79 ± 0.93	61 ± 0.6	55.26	5.53	5.33	0.96	-	∼2 h

Note:.

⁎ The cost of PEG 6000 and PEG 8000 are similar from most vendors. % purity and total protein recovery are mean ± SD of three independent experiments.

PEG had been promoted as a valuable reagent for IgY extraction because it is non-toxic, chemically inactive, stable even at high temperatures and concentrations, and does not alter the natural interactions between proteins (Annunziata et al., 2002). Considering the LLPS principle, an additional advantage of PEG is that it retains the native antibody conformation (Bramham et al., 2021). This property, couple with the fact that the final IgY is precipitated as a pellet, during the second centrifugation (third when using PEG 6000), allows the use of extracted IgY directly without the need for the removal of traces of PEG (Goldring and Coetzer, 2003). With other precipitants such as ammonium sulfate, dialysis is necessary before the IgY can be used.

### The novel method offer advantages

**Fig. 4:**
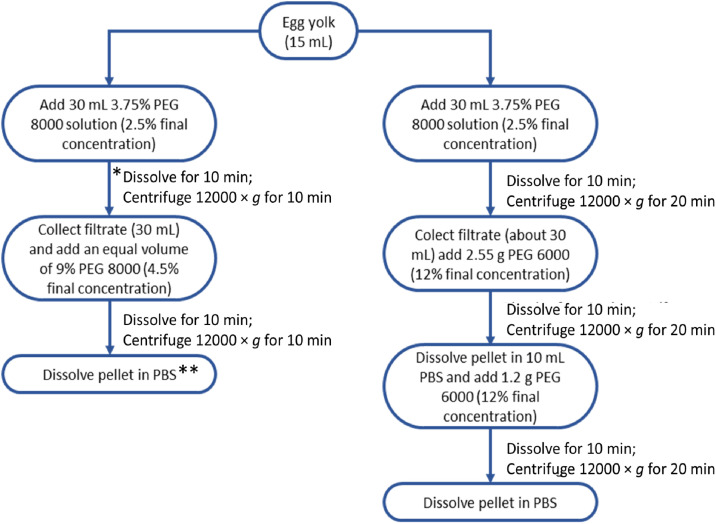
**Protocols for the extraction of IgY from egg yolk with PEG 8000 or PEG 6000. Notes:** Starting from 15 mL egg yolk each, the amount of PEG 8000 and PEG 6000 required to achieve IgY extraction is 3.82 g and 5.33 g respectively. The PEG 6000 method is as described by Pauly et al. (2011). *The incubation time at this stage is very critical as prolonged incubation may lead to irreversible formation of aggregates resulting in inactive IgY. **The precipitate produced is colorless, hence the tube must be washed thoroughly and adequately to recover the maximum amount of IgY.

PEG induces protein precipitation by exerting a depletion force on the protein, a property that increases with the increasing molecular weight of PEG. During IgY extraction, PEG 8000 achieved the two key requirements of IgY extraction from egg yolk, viz, delipidation to remove egg yolk lipids and enriching the IgY content of the protein fraction (Chang et al., 2018; Morgan et al., 2021) after two centrifugation steps (Fig. 4). Furthermore, PEG 8000 demonstrated these effects at lower concentrations compared to PEG 6000. The advantages of PEG 6000, such as safety and low cost, are retained with PEG 8000, and we demonstrated here that the novel method produces IgY with similar purity and antigen binding properties compared to PEG 6000 (Fig. 2, Fig. 3). The new method achieves IgY extraction in two steps, significantly reducing the amount of PEG used, leading to cost savings of up to 40 % compared to conventional PEG precipitation. This added an additional dimension to the advantage of PEG 8000 as a protein extraction medium (Table 1). Furthermore, in a laboratory setting, a typical immunization experiment involves collecting eggs over several weeks (usually about 12-16 weeks) resulting is over a 100 eggs/hen. Thus, time becomes a factor when purifying IgY from large number of eggs, possibly across multiple experiment. In the PEG6000 method, the PEG powder is added to the yolk dilution (as the solution obtained subsequently), which increase the waiting time. As the dissolution rate of PEG varies with temperature (D'Souza A and Shegokar, 2016), the duration for the complete PEG 6000 dissolution may be even more prolonged under certain conditions. By starting with PEG 8000 masterbatch solution, our method ensures a more consistent PEG solution and reduces the wait time. Thus, this novel method is particularly valuable for time saving when purifying IgY from a large number of eggs.

Our proposed IgY extraction method offers a promising alternative but has limitations that need addressing. Primarily, it yields less IgY compared to conventional methods, as the use of PEG 8000 prioritizes purity and reduces aggregation risks through LLPS, which lowers protein recovery relative to PEG 6000. However, this lower yield is typically a concern only in cases requiring maximum recovery per immunization schedule. Additionally, contaminating proteins are present in the final IgY solution, necessitating further purification for high-purity applications. Some of these contaminants, like vitellogenin II precursor fragments, co-precipitate with IgY under several conditions (Almeida et al., 2022; Klimentzou et al., 2006). While this method reduces PEG 8000 use and achieves significant cost savings, balancing cost-effectiveness with desired yield and purity will be essential for refining the method's utility in large-scale IgY production for biomedical applications.

## Conclusion

Utilizing the protein LLPS principle with PEG 8000 presents an efficient and innovative approach for IgY extraction from egg yolk, delivering purity comparable to existing methods. Unlike other polymer-based techniques, this method avoids IgY aggregation, making it particularly valuable for studies focused on the physicochemical properties of IgY where aggregates could interfere. Additionally, this method offers significant advantages in terms of reduced costs and time, further enhancing its utility especially for research applications.

## Disclosures

The authors declare no conflict of interest.
